# Urinary biomarkers as indicator of chronic inflammation and endothelial dysfunction in obese adolescents

**DOI:** 10.1186/s40608-017-0148-2

**Published:** 2017-03-22

**Authors:** Ruchi Singh, Arushi Verma, Salim Aljabari, Tetyana L. Vasylyeva

**Affiliations:** Department of Pediatrics, Texas Tech University Health Sciences Center, 1400 S. Coulter, Amarillo, TX 79106 USA

**Keywords:** Inflammatory markers, Urinary biomarkers, Obesity, Chronic inflammation

## Abstract

**Background:**

Obesity is a pro-inflammatory state that may predispose patients to acute coronary syndrome characterized by chronic low grade inflammation resulting in endothelial dysfunction (ED). The aim of the study was to evaluate urinary biomarkers of inflammation and ED in adolescents with obesity.

**Methods:**

Sixty three subjects were recruited for the study. Twenty healthy adolescents with normal body mass (NW), 14 overweight (OW), 29 obese (OA) subjects were selected. An EndoPat 2000 device was used to measure the reactive hyperemia index (RHI). First morning fasting urine samples were tested for interleukin 6 (IL-6), endothelin 1 (ET-1), alpha-1-acid glycoprotein (AGP), tumor necrosis factor- α (TNF-α) and corrected to urinary creatinine.

**Results:**

Urinary TNF-α was significantly higher in OA group (52.4 ± 15.3 pg/mg) compared to adolescents with NW (14.1 ± 1.2 pg/mg, *P* = 0.04). ET-1 levels were found to be higher in OW (5.18 ± 1.6 pg/mg) compared with NW (3 · 47 ± 0.3 pg/mg, *P* = 0.24); and higher in OA (8.48 ± 3.1 pg/mg) compared to both NW (*P* = 0.19) and OW (*P* = 0.40). Similarly a higher AGP level was observed in OW (864.8 ± 156 ng/mg) and OA (808.3 ± 186 ng/mg) compared to NW (653 ± 69 ng/mg) (*P* = 0.16 & 0.49 respectively). Inflammatory markers namely, TNF-α, IL-6 and AGP significantly and positively correlated with each other and with ET-1, a marker for endothelial dysfunction. This significant correlation was also observed when tested separately in the subgroups (NW, OW and OA). There were no differences in RHI levels among the study groups.

**Conclusion:**

Urinary TNF-alpha is significantly elevated in obese adolescents and correlates with urinary ET-1, which is recognized as a biomarker for endothelial dysfunction. Since obesity is a chronic inflammatory state, elevated urinary TNF-alpha might be used as a non invasive tool to monitor the level of that inflammation.

## Background

Obesity is well known to be major risk factor for the early development of atherosclerosis and consequently cardiovascular complications [1, 2]. The prevalence of this condition among children and adolescents has been growing at an alarming rate over the last two decades resulting in huge burden on health care system [1–4].

From 9 to 20% of American children and adolescents are obese. They are likely to be obese in adulthood and develop obesity-related health problems (https://www.cdc.gov/obesity/data/childhood.html). Obesity has been pointed out as a contributing factor to approximately 100,000–400,000 deaths in the United States per year [5].

Obesity beginning at an early age in childhood increases the morbidity associated with it and creates the tendency for increased risk of chronic diseases such as cardiovascular disease (CVD). This increased risk of CVD in obesity has traditionally correlated with major risk factors such as diabetes, hypertension, and hyperlipidemia [6, 7]. These major risk factors are validated in many populations [8–10] for the diagnosis and management of CVD. The underlying mechanisms for the association of obesity and major risk factors with CVD are still not fully understood, but it seems that chronic low grade inflammation plays a substantial role.

On the other hand, formation of atherosclerosis is a slow process and starts years before the clinical manifestation. The process starts as early as in the first and second decades of life in individuals at risk and manifests usually after the third to fourth decades of life. This fact motivated the clinical researchers over the years to develop a valid screening test for the early detection and an intervention to abort or at least slow the atherosclerosis formation before it becomes an irreversible condition [6–8].

It was established that endothelial dysfunction (ED) represents the early reversible stage in the development of atherosclerosis. Both obesity and diabetes mellitus were found to be strongly associated with ED and the subsequent atherosclerosis formation [9–12].

Chronic low-grade subclinical systemic inflammation was found in both diabetes and obesity. Those patients have high serum levels of inflammatory cytokines and other markers such as IL-6, TNF- α, C-reactive protein, AGP and few others. The elevated serum levels of inflammatory markers were strongly associated with deterioration in the endothelial function in obese and diabetic individuals, suggesting that diabetes and obesity represent a state of chronic systemic inflammation that in turn cause ED [13–16]. Multiple studies showed that reactive hyperemia index (RHI) is good noninvasive indicator of endothelial dysfunction [17–21].

The use of urinary biomarkers as a noninvasive screening test for systemic diseases outcomes monitoring has gained interest recently. Urinary biomarkers in obese, metabolically deranged and type 2 diabetes have significantly correlated with kidney disease and vascular damage in the adult population [22, 23]. Children and adolescents represent a population subgroup where such a noninvasive approach is the most desirable [24]. While teenagers are old enough to refuse painful procedures, most of time they are not mature enough to understand the importance of routine screening tests [24]. Whereas serum inflammatory markers have been studied in the obese pediatric population [25–28], there is paucity of data on urinary markers in the non-diabetic, overweight and obese adolescent population.

The goal of the current study was to investigate urinary biomarkers of ED and inflammation in overweight and obese adolescents to provide a basis for non-invasive screening and monitoring. We also compared endothelin 1 (ET-1), urinary biomarker of ED, with the previously standardized noninvasive test RHI.

## Methods

### Study population

The study was approved by local IRB and informed consent was obtained from all subjects before enrollment. Healthy with within normal limit weight (NW), overweight (OW) and obese adolescents (OA) were recruited from Texas Tech University Health Science Center (TTUHSC) pediatric clinics. CDC growth charts for the body mass index (BMI) were used to classify the participants into obese (BMI > 95%), overweight (BMI 85–95%) and subjects with normal weight (BMI < 85%). Adolescents from 10 to 20 years old were enrolled in the study. Both informed consent and assent were obtained from the legal guardian and the participant, who were younger than 18 years old after explaining the goals of the study and the potential risks. Informed consents were obtained for participants 18 years and older. At the start of the study, anthropometric (body height and weight), biological (blood pressure, heart rate and physical fitness), lifestyle (dietary habits, smoking behavior, and daily physical activity), and psychological variables were assessed. In addition, first morning fasting urine samples of the participants were collected, and biomarker of endothelial dysfunction and four biomarkers of low-grade inflammation were measured.

### Assessment of endothelial dysfunction and low-grade inflammation

Urinary biomarkers of endothelial dysfunction (ET-1), and of low-grade inflammation (c-reactive protein (CRP), alpha-1-acid glycoprotein (AGP), tumor necrosis factor-α (TNF-α), and interleukin-6 were assessed by ELISA (R&D Systems, Minneapolis, MN) per manufacture instructions. All markers were normalized with urinary creatinine by creatinine ELISA kit (R&D Systems, Minneapolis, MN).

### Reactive hyperemia index (RHI)

The Endo-PAT 2000 (Itamar 2000) machine was used in measuring the RHI. Each participant was placed in a quiet room with dim light and led to rest for 20 min before starting the procedure. The Endo-PAT probe was placed on the index finger and a blood pressure cuff was placed on the arm. A baseline reading was recorded for 5 min then blood pressure cuff was inflated to 40 mm Hg above the systolic blood pressure for 5 min. Following this the machine recorded the readings for 10 min and the Endo-PAT software calculated RHI from the two readings.

### Other measurements

Weight, height, body mass index, heart rate, and blood pressure were determined according to recommended CDC standards (https://www.cdc.gov/growthcharts/clinical_charts.htm).

### Statistical analysis

Microsoft Excel spreadsheet to maintain the data was used. For each parameter group means are presented with the standard error of the mean as the index of dispersion. Statistical significance was assessed using analysis of variance followed by t-tests. Pearson’s correlation was calculated between various study parameters. Values of ‘p’ less than or equal to 0.05 were considered statistically significant. Variables with a skewed distribution (CRP, interleukin 6, TNF alpha, ET-1 and AGP) were ranked using nonparametric Wilcoxon-Whitney analysis. Overall *z* scores were calculated for ED or low-grade inflammation. We also calculated Receiver operating characteristics (ROC) by Winnonlin software.

## Results

Sixty three adolescents of 10–20 years age were included in the study. There were 20 subjects in healthy normal BMI group (NW), 14 subjects in overweight group (OW), 29 subjects in obese group (OA) (Table 1).

**Table 1:** Age of participants

	Male (*n* = 32)	Female (*n* = 31)	Total	Age
NW	12	8	20	13.9 ± 2.0
OW	7	7	14	13.5 ± 2.4
OA	13	16	29	14.2 ± 2.5
Average				13.8 ± 2.3

Analysis of dietary habits with *t*-test shows that there was no significant difference in carbohydrate, fat or protein consumption between normal, overweight and obese adolescents. However obese male adolescents consumed significantly more carbohydrates (average 4.07 ± 3.24 servings/day) as compared to obese female adolescents (2.04 ± 1.07 servings/day), *P* = 0.039 (Table 2).

**Table 2:** Dietary distribution of participants

	Avg. carb serving/day	Avg. fat serving/day	Avg. protein serving/day
NW Males	2.18 ± 0.92^*^	0.84 ± 0.39	2.65 ± 0.94
NW Females	2.0 ± 0.46	1.6 ± 0.84	2.18 ± 1.09
OW Males	1.75 ± 0.5	1.5 ± 0.7	1.83 ± 1.63
OW Females	2.5 ± 1.52	1.83 ± 1.52	2.16 ± 1.25
OA Males	4.07 ± 3.24^*, **^	0.91 ± 0.66	2.93 ± 1.29
OA Females	2.04 ± 1.07^**^	1.54 ± 0.79	2.34 ± 0.80

*NA* Normal weight, *OW* Overweight, *OA* Obese; Data is presented as Mean ± SD

^*,**^ *P* < 0.05

**Fig. 1:**
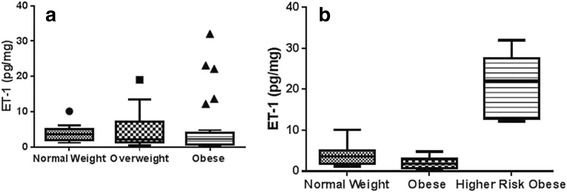
**a** Comparison of median (min–max) urinary ET-1 (pg/mg) among normal weight (*n* = 20), overweight (*n* = 14) and obese (*n* = 29) group. Data was compared by using tukey’s test. Data is represented as *Whisker-box plot* and *outliers* are plotted as individual points. **b** Median (min–max) urinary ET-1 (pg/mg) among normal weight (*n* = 20), overweight (*n* = 14) and high risk obese (*n* = 17) group

**Fig. 2:**
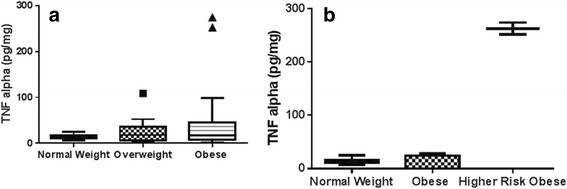
**a** Comparison of median (min–max) urinary TNF-α (pg/mg) among normal weight (*n* = 20), overweight (*n* = 14) and obese (*n* = 29) group. Data was compared by using tukey’s test in combination with post-hoc analysis. Data is represented as *Whisker-box plot* and *outliers* are plotted as individual points. **b** Median (min–max) urinary TNF-α (pg/mg) among normal weight (*n* = 20), overweight (*n* = 14) and high risk obese (*n* = 17) group

**Fig. 3:**
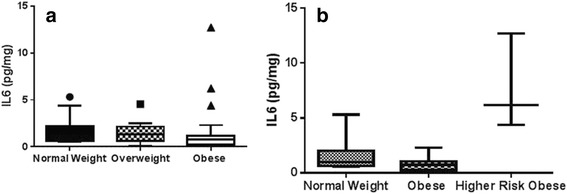
**a** Comparison of median (min–max) urinary IL-6 (pg/mg) among normal weight (*n* = 20), overweight (*n* = 14) and obese (*n* = 29) group. Data was compared by using tukey’s test in combination with post-hoc analysis. Data is represented as *Whisker-box plot* and *outliers* are plotted as individual points. **b** Median (min–max) urinary IL-6 (pg/mg) among normal weight (*n* = 20), overweight (*n* = 14) and high risk obese (*n* = 17) group

**Fig. 4:**
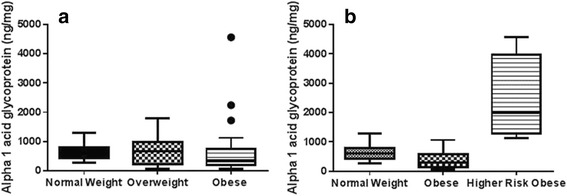
**a** Comparison of median (min–max) urinary AGP (ng/mg) among normal weight (*n* = 20), overweight (*n* = 14) and obese (*n* = 29) group. Data was compared by using tukey’s test in combination with post-hoc analysis. Data is represented as *whisker-box plot* and *outliers* are plotted as individual points. **b** Median (min–max) urinary AGP (ng/mg) among normal weight (*n* = 20), overweight (*n* = 14) and high risk obese (*n* = 17) group

Obese males also consumed significantly more carbohydrates (average 4.07 ± 3.24 servings/day) as compared to normal weight male adolescents (average 2.18 ± 0.92), *P* = 0.04, but there was no difference between carbohydrate consumption of normal weight and obese females. With respect to clinical characteristics, obese adolescents had a higher systolic BP percentile (average 77th ± 32.0 percentile) as compared with normal weight adolescents (average 59th ± 19.8 percentile), *P* = 0.01 using *t*-test statistical analysis. There was no significant difference in heart rate among the three groups of adolescents. No statistical significance was seen in ET-1 among OW (5.18 ± 1.6 pg/mg) as compared to NW (3.47 ± 0.3 pg/mg, *P* = 0.24) and among OA (8.48 ± 3.1 pg/mg) as compared to NW (*P* = 0.19) and OW (*P* = 0.40) (Fig. 1). TNF-α was significantly higher in OA (52.4 ± 15.3 pg/mg) compared to NW (14.1 ± 1.2 pg/mg, *P* = 0.04) (Fig. 2). IL-6 levels were no different in OW (13.8 ± 3.2 pg/mg) or in OA (15.8 ± 4.6 pg/mg) compared to NW (15.3 ± 2.9) (*P* = 0.74, 0.93 respectively) (Fig. 3). Again, no differences in AGP levels were seen in OW (864.8 ± 156 ng/mg) and OA (808.3 ± 186 ng/mg) compared to NW (653 ± 69 ng/mg) (*P* = 0.16, 0.49 respectively) (Fig. 4).

There was a group of obese individuals (58% of all OA, *n* = 17) who had at least one inflammatory marker more than one standard deviation above the standard mean (Table 3). This group was segregated by Tukey’s test, which identifies any difference between two means that is greater than the expected standard error. We identified this as a ‘high risk’ obese group (HA) (Table 3).

**Table 3:** High risk obese group

All obese (*n* = 29)	Percentage of OA with ET-1 > 11.5 pg/mg	Percentage of OA with TNF-α >67.6 pg/mg	Percentage of OA with IL-6 > 20.4 pg/mg	Percentage of OA with AGP >994.4 pg/mg
Males	14% (*n* = 4)	7% (*n* = 2)	7% (*n* = 2)	10% (*n* = 3)
Females	3% (*n* = 1)	3% (*n* = 1)	3% (*n* = 1)	7% (*n* = 2)
Total	17% (*n* = 5)	10% (*n* = 3)	10% (*n* = 3)	17% (*n* = 5)

*OA* Obese, *ET-1* endothelin-1, *TNF-α* tumor necrosis factor-alpha, *IL-6* interleukin-6, *AGP* alpha-1 acid glycoprotein

**Fig. 5:**
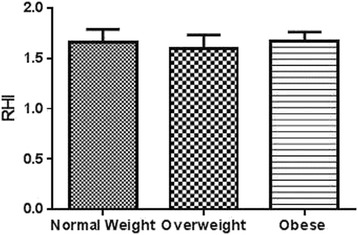
Comparison of mean (±SD) reactive hyperemia index (RHI) among normal weight (*n* = 20), overweight (*n* = 14) and obese (*n* = 29) group by *t*-test

Ten percent of these high risk obese adolescents (*n* = 3) had elevation of all four inflammatory markers, namely urinary ET-1, TNF-α, IL-6 and AGP. Of these, two were males and one female. Upon comparing high risk obese group individuals with NW and OW, ET-1, TNF-α, IL-6 and AGP were found to be significantly higher (Figs. 1b, 2b, 3b and 4b). No difference was observed in the RHI compared with OW (1.66 ± 0.1) and NW (1.60 ± 0.1, *P* = 0.78) or OA (1.67 ± 0.09) and HA (*P* = 0.65) (Fig. 5).

**Fig. 6:**
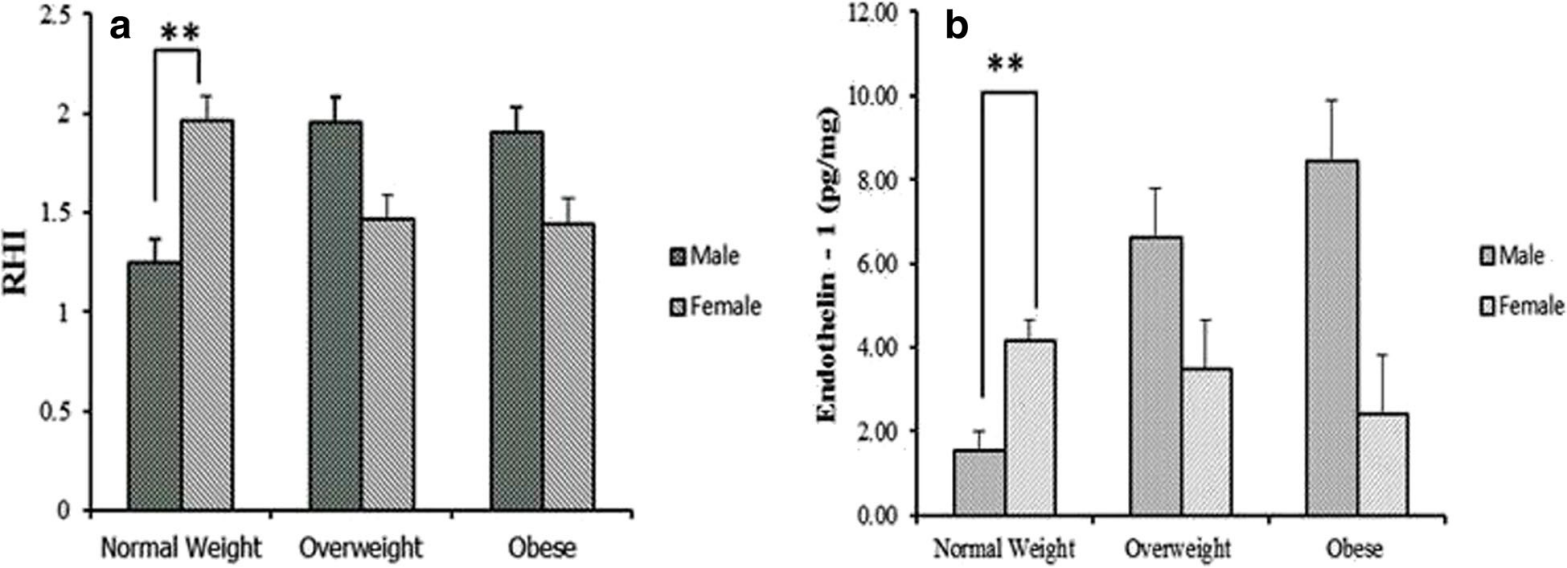
**a** Sex based comparison of Reactive Hyperemia Index (RHI) (mean ± SD) among normal weight (*n* = 20), overweight (*n* = 14), and obese groups (*n* = 29) (**P <0.01). **b** Sex based comparison of ET-1 (mean ± SD) for endothelin 1 (ET-1) normal weight (*n* = 20), overweight (*n* = 14), and obese groups (*n* = 29) (**P <0.01)

There was no statistically significant gender difference in the urinary biomarkers of inflammation (urinary TNF-α and urinary IL-6). However, sex difference was observed in RHI values in healthy population with significantly higher index in female compared to male (Fig. 6a). ET-1 was also found significantly higher in healthy female as compared to their male peers (Fig. 6b).

**Fig. 7:**
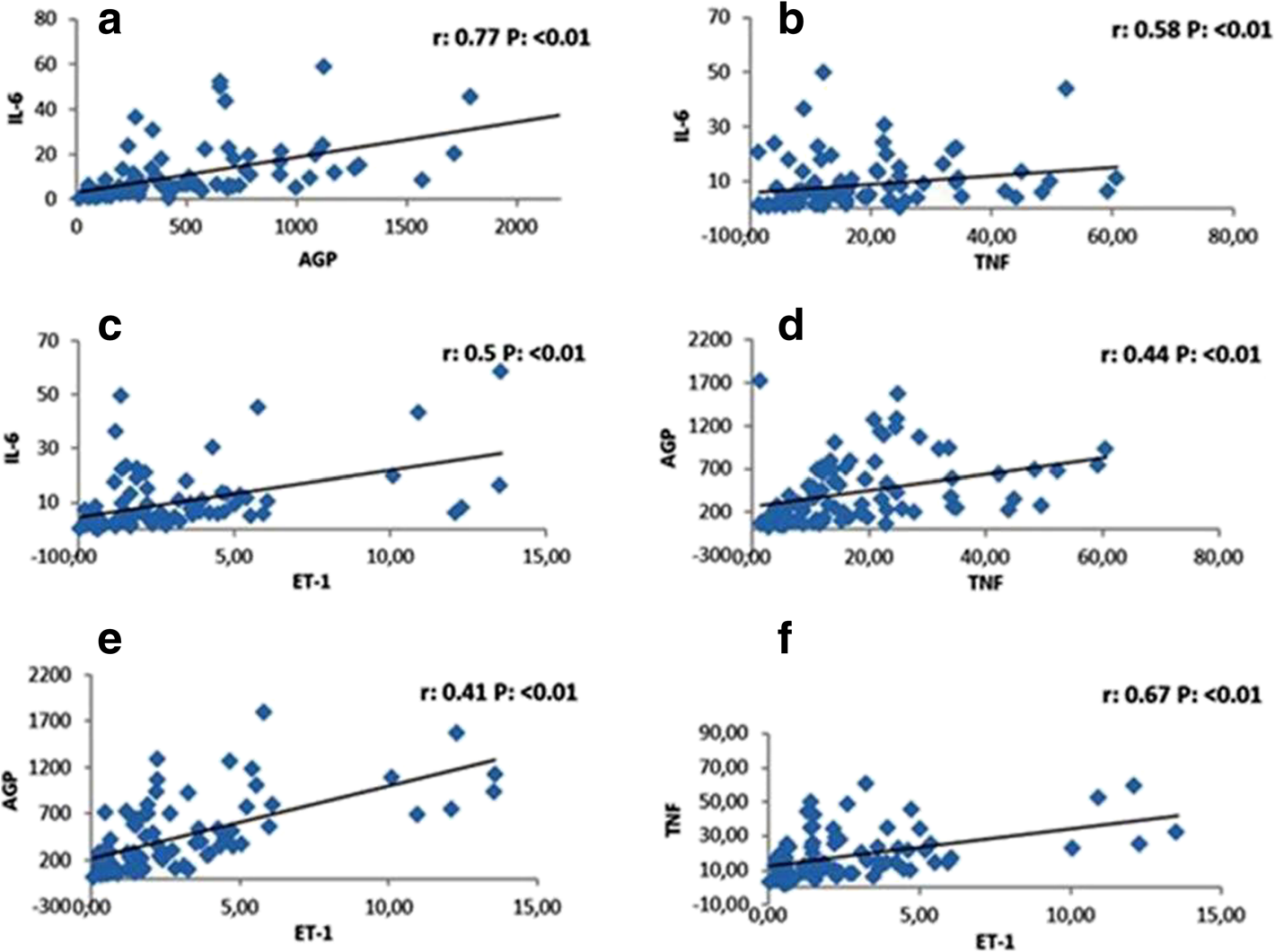
Correlation between the different urinary biomarkers irrespective of age, weight and gender. *N* = 63. **a** Correlation of interleukin (IL-6) and alpha 1 acid glycoprotein (AGP); **b** Correlation of interleukin (IL-6) and tumor necrosis factor alpha (TNF-α); **c** Correlation of interleukin (Il 6) and endothelin 1 (ET-1); **d** Correlation of alpha 1 acid glycoprotein (AGP) and tumor necrosis factor alpha (TNF-α); **e** Correlation of alpha 1 acid glycoprotein (AGP) and endothelin 1 (ET-1); **f** Correlation of tumor necrosis factor alpha (TNF α) and endothelin 1 (ET-1)

As shown in Fig. 7, the four inflammatory markers were significantly and positively correlated. Significant correlation was also observed when tested separately in the subgroups (NW, OW, and OA). However RHI did not show any significant correlation with inflammatory markers (TNF-α r: 0.07, *P* = 0.50; IL-6 r: −0.07, *P* = 0.49; ET-1 r: −0.02, *P* = 0.78; AGP r: −0.05 *P* = 0.58) (Fig. 7).

## Discussion

Although measurement of reactive hyperemia was advocated for an adult population as a sensitive marker of endothelial dysfunction [29, 30], in our study we did not find significant difference in the RHI between obese and adolescents with normal weight. In fact, the RHI values in three groups were very close. Though RHI did not show any significant correlation with the urinary inflammatory markers, it could be possible that changes in endothelial function as measured by RHI, is a late finding in the inflammation to obesity to cardiovascular disease development. Interestingly, we observed sex based difference of this index in teenagers. Gender-related differences in healthy adults have already been studied. The Framingham Heart Study reported a lower PAT hyperemic response in males compared to females, likely because estrogens stimulate NO synthesis [31].

While this parameter is thought to be very sensitive in the detecting ED in adults, our results question its sensitivity in adolescents. Most likely, endothelial function in obese adolescents is not compromised enough to produce significant difference on the finger plethysmography. Thus this method, though non-invasive and convenient, is not likely to be the first choice to monitor the early changes in endothelial function.

Endothelin-1 is also a marker of endothelial dysfunction and potent vasoconstrictor, which is produced by vascular endothelial cells. Urinary ET-1 earlier was found to be elevated in adolescents with insulin dependent diabetes and was positively correlated with microalbuminuria [32, 33]. Interestingly in adolescents with diabetes, serum ET-1 was not found to be as sensitive [33] as other authors determined it in urine [32].

In our study, ET-1 showed tendency to be higher in OA, although it was not statistically significant. But this observation should not be ignored due to the fact that ET-1 showed significant and strong correlation with urinary markers of inflammation, particularly TNF-α.

Analyzing urinary ET-1 is less time consuming for patients and more cost efficient with comparable sensitivity and specificity to RHI. We also observed sex differences in levels of urinary ET-1 with the higher number in adolescent girls. Looking for an explanation of this finding, we found that a previous adult study addressed the sex difference in serum ET-1. Polderman et al. showed that male hormones may increase plasma endothelin levels, whereas female hormones can lower them [32]. Another study showed contradictory results, with plasma ET-1 levels significantly higher in women than in men (7.1 ± 3.2 vs 4.5 ± 3.6; *p* < 0.01) [34]. It has been reported that ET-1 is induced by agents known to modulate calcium [35]. Thus estrogen would theoretically increase ET-1 levels [34]. We also observed similar findings in our study with urinary excretion of ET-1. AGP inflammatory marker significantly and positively correlated with ET-1 and TNFα. This significant correlation was also observed in different subgroups based on BMI. The serum concentration of AGP rises two to five times during an acute phase response and changes its glycosylation pattern depending on the type of inflammation [36]. The urinary level of TNF-α in obese adolescents was significantly higher than counterparts with normal weight and even in comparison to overweight adolescents. TNF-α is an inflammatory marker that is well proved to be high in obese adult individuals reflecting the subclinical systemic inflammation. Obese teens have been shown to have chronic inflammation and ED, by elevation of different serum markers [14, 17]. This study showed for the first time that in comparison with the other urinary markers, TNF-α is the most sensitive marker to weight related body disorders.

Although we did not find a significant increase in the other inflammatory markers in the urine of OA, namely the IL-6 and orosomucoid, both showed tendency to be higher and had a significant positive correlation with TNF-α. A larger study with serum and urine levels might be needed to accurately test the sensitivity of IL-6 and orosomucoid as urinary biomarkers for systemic inflammation. In 2002 Wallenius found that IL-6 was increased in obesity and responsible for insulin resistance [37]. However the effect of IL-6 production involvement in alteration in inflammatory signal is not fully known [37]. The reason of gender difference and expression of TNF alpha and IL-6 needs to be further investigated. One reason may be that the most of the human studies performed so far are cross-sectional studies or are case control studies. There is need for prospective assessment of these biomarkers. In present study, we found variability in IL-6 values in obese group.

## Conclusion

Urinary TNF-alpha is significantly elevated in obese adolescents and correlates with urinary ET-1, which is recognized as a biomarker for endothelial dysfunction. Since obesity is a chronic inflammatory state, elevated urinary TNF-alpha might be used as a non invasive tool to monitor the level of that inflammation. Urinary ET-1 is as sensitive test for ED as RHI. Sex differences exist in healthy adolescents with normal weight in levels of urinary ET-1 and RHI, which is blunted in overweight and obese individuals.

## Abbreviations

AGP: Alpha-1-acid glycoprotein
BMI: Body mass index
CDC: Center for Disease Control
CRP: C-reactive protein
CVD: Cardiovascular disease
ED: Endothelial dysfunction
ELISA: Enzyme-linked Immunosorbent Assay
ET-1: Endothelin-1
HA: High risk obese
IL-6: Interleukin-6
NO: Nitric oxide
NW: Normal weight
OA: Obese
OW: Overweight
RHI: Reactive hyperemia index
ROC: Receiver operating characteristics
TNF alpha: Tumor necrosis factor alpha
TTUHSC: Texas Tech University Health Sciences Center

## References

[1] Lobstein T, Jackson-Leach R (2007). Child overweight and obesity in the USA: prevalence rates according to IOTF definitions. Int J Pediatr Obes.

[2] Ogden CL, Carroll MD, Kit BK, Flegal KM. Prevalence of obesity in the United States, 2009–2010. NCHS Data Brief. 2012;(82):1–8. Centers for Disease Control and Prevention National Center for Health Statistics: 2009–2010.

[3] Hotu S (2004). Increasing prevalence of type 2 diabetes in adolescents. J Paediatr Child Health.

[4] Hsia Y (2009). An increase in the prevalence of type 1 and 2 diabetes in children and adolescents: results from prescription data from a UK general practice database. Br J Clin Pharmacol.

[5] Blackburn GL, Walker WA (2005). Science-based solutions to obesity: what are the roles of academia, government, industry, and health care?. Am J Clin Nutr.

[6] Kimura BJ (2003). Detection of early carotid arterial atherosclerosis by briefly trained physicians using a hand-held ultrasound device. Am J Cardiol.

[7] van den Oord SCH (2013). Assessment of subclinical atherosclerosis and intraplaque neovascularization using quantitative contrast-enhanced ultrasound in patients with familial hypercholesterolemia. Atherosclerosis.

[8] Caballero P (2012). Detection of subclinical atherosclerosis in familial hypercholesterolemia using non-invasive imaging modalities. Atherosclerosis.

[9] Mudau M (2012). Endothelial dysfunction: the early predictor of atherosclerosis. Cardiovasc J Afr.

[10] Yang Z, Ming XF (2006). Recent advances in understanding endothelial dysfunction in atherosclerosis. Clin Med Res.

[11] Meyers MR, Gokce N (2007). Endothelial dysfunction in obesity: etiological role in atherosclerosis. Curr Opin Endocrinol Diabetes Obes.

[12] Sitia S (2010). From endothelial dysfunction to atherosclerosis. Autoimmun Rev.

[13] Ikonomidis I (2008). Inflammatory and non-invasive vascular markers: the multimarker approach for risk stratification in coronary artery disease. Atherosclerosis.

[14] Montero D (2012). Endothelial dysfunction, inflammation, and oxidative stress in obese children and adolescents: markers and effect of lifestyle intervention. Obes Rev.

[15] Siervo M (2012). Body mass index is directly associated with biomarkers of angiogenesis and inflammation in children and adolescents. Nutrition.

[16] Giordano P (2011). Metabolic, inflammatory, endothelial and haemostatic markers in a group of Italian obese children and adolescents. Eur J Pediatr.

[17] Giannini C (2009). Increased carotid intima-media thickness in pre-pubertal children with constitutional leanness and severe obesity: the speculative role of insulin sensitivity, oxidant status, and chronic inflammation. Eur J Endocrinol.

[18] Kapiotis S (2006). A proinflammatory state is detectable in obese children and is accompanied by functional and morphological vascular changes. Arterioscler Thromb Vasc Biol.

[19] Jimenez MV (2007). Endothelial dysfunction is related to insulin resistance and inflammatory biomarker levels in obese prepubertal children. Eur J Endocrinol.

[20] Meyer AA (2006). Impaired flow-mediated vasodilation, carotid artery intima-media thickening, and elevated endothelial plasma markers in obese children: The impact of cardiovascular risk factors. Pediatrics.

[21] Jiang HJ (2009). Increased urinary excretion of orosomucoid is a risk predictor of diabetic nephropathy. Nephrology.

[22] Satoh-Asahara N (2011). Urinary Cystatin C as a Potential Risk Marker for Cardiovascular Disease and Chronic Kidney Disease in Patients with Obesity and Metabolic Syndrome. Clin J Am Soc Nephrol.

[23] Eynatten M (2009). Urinary adiponectin excretion: a novel marker for vascular damage in type 2 diabetes. Diabetes.

[24] Rizvi AA (2009). Cytokine Biomarkers, Endothelial Inflammation, and Atherosclerosis in the Metabolic Syndrome: Emerging Concepts. Am J Med Sci.

[25] Silva LR, Stefanello JM, Pizzi J, Timossi LS, Leite N (2012). Atherosclerosis subclinical and inflammatory markers in obese and nonobese children and adolescents. Rev Bras Epidemiol.

[26] Wang H, Steffen LM, Vessby B, Basu S, Steinberger J, Moran A, Jacobs DR, Hong CP, Sinaiko AR (2011). Obesity modifies the relations between serum markers of dairy fats and inflammation and oxidative stress among adolescents. Obesity (Silver Spring).

[27] Valle Jiménez M, Estepa RM, Camacho RM, Estrada RC, Luna FG, Guitarte FB (2007). Endothelial dysfunction is related to insulin resistance and inflammatory biomarker levels in obese prepubertal children. Eur J Endocrinol.

[28] Chang C-J (2015). Evidence in Obese Children: Contribution of Hyperlipidemia, Obesity-Inflammation, and Insulin Sensitivity. PLoS ONE.

[29] Binggeli C, Noll G (2004). Reactive hyperemia as a test of endothelial or microvascular function? Reply. J Am Coll Cardiol.

[30] Minson CT, Wong BJ (2004). Reactive hyperemia as a test of endothelial or microvascular function?. J Am Coll Cardiol.

[31] Lantin Hermoso RL (1997). Estrogen acutely stimulates nitric oxide synthase activity in fetal pulmonary artery endothelium. Am J Phys Lung Cell Mol Phys.

[32] Polderman KH (1993). Influence of Sex-Hormones on Plasma Endothelin Levels. Ann Intern Med.

[33] Evans RR (1996). Racial and gender differences in endothelin-1. Am J Cardiol.

[34] Maggi M, Vannelli GB, Peri A, Brandi ML, Fantoni G, Giannini S, Torrisi C, Grrardabasso V, Bami T, Toscano V (1991). Immunolocalization, binding, and bio-logical activity of endothelin in rabbit uteras: effect of ovarian steroids. Am J Physiol..

[35] Hamburg NM (2008). Cross-sectional relations of digital vascular function to cardiovascular risk factors in the Framingham Heart Study. Circulation.

[36] Hochepied T (2003). alpha(1)-Acid glycoprotein: an acute phase protein with inflammatory and immunomodulating properties. Cytokine Growth Factor Rev.

[37] Wallenius K (2002). Intracerebroventricular interleukin-6 treatment decreases body fat in rats. Biochem Biophys Res Commun.

